# Severe war-related trauma and personality pathology: a case-control study

**DOI:** 10.1186/s12888-017-1269-3

**Published:** 2017-03-21

**Authors:** Jasna Munjiza, Dolores Britvic, Maja Radman, Mike J. Crawford

**Affiliations:** 1 Centre for Psychiatry, Faculty of Medicine, Imperial College London, Hammersmith Campus, 7th Floor Commonwealth Building, Du Cane Road, London, W12 0NN UK; 2 Central and North West London NHS Foundation Trust, London, UK; 3 Department of Psychiatry, School of Medicine Split, University of Split, Split, Croatia; 4 Department of Internal Medicine, School of Medicine Split, University of Split, Split, Croatia

**Keywords:** War, Severe trauma, PTSD, Personality pathology

## Abstract

**Background:**

Exposure to war-related trauma has long been recognised to have an adverse effect on mental health. We attempted to investigate whether people who have clinically significant personality-related problems 15 years after a war are more likely to have been exposed to severe war-related trauma than those who do not have significant personality difficulties.

**Methods:**

A case –control study was conducted in southern Croatia, fifteen years after the 1991–1995 war. We recruited 268 participants: 182 cases who scored positively on the International Personality Disorder Examination scale (IPDE), and 86 controls who were IPDE negative. Severity of war-related trauma was assessed according to the 17 items on the Harvard Trauma Questionnaire (HTQ) trauma event scale, which were considered to be of severe (catastrophic) nature based on the ICD-10 description of catastrophic trauma and the opinion of trauma experts. All participants also completed measures of mental health (depression, anxiety and PTSD), social functioning and current substance misuse.

**Results:**

Cases (IPDE positive) were eight times more likely to report exposure to severe war-related trauma than controls. This association increased after adjustments for demographic factors (OR = 10.1, 95% CI 5.0 to 20.4). The types of severe trauma most frequently reported were either the participants’own life being in direct danger or witnessing extreme violence inflicted on others or the result of violence towards others (murder, torture, seeing burned or disfigured bodies). Prevalences of depression, anxiety and PTSD were high among IPDE positive participants 15 years after exposure to war trauma. Their level of interpersonal dysfunction was considerably higher than that in controls (OR = 10.39, 95% CI 3.51 to 30.75). Alcohol consumption in cases was significantly higher with a mean of 14.24 units per week (sd = 11.03) when compared to controls whose mean number of alcohol units was 9.24 (sd = 7.25), t (73) = 2.16, *p* < 0.05, mean difference 4.99 (95% CI = 0.39 to 9.60). Similarly, a significantly higher number of cases reported current substance misuse (8.2% vs. 0.0%) X^2^ (1, *n* = 268) = 7.51, *p* < 0.05).

**Conclusion:**

Exposure to severe war-related trauma is a risk factor for interpersonal dysfunction15 years after people were exposed to an armed conflict. These findings have implications for assessing and meeting the long-term mental health needs of people in war-affected regions. Further research needs to be done to increase our understanding about the relationship between severe war trauma and personality related problems.

## Background

War can have a detrimental effect on people’s physical and mental health. This is not only related to adversities resulting from people losing their homes and being displaced or being an indirect consequence of destroyed infrastructure, but it is also associated with direct exposure to interpersonal violence (murder, torture or being exposed to other life threatening situations). High levels of mental distress, depression, anxiety and post-traumatic stress disorder (PTSD) have been reported in the studies conducted with people exposed to war trauma [1, 2]. People suffering from PTSD have higher levels of comorbidity (both mental and physical illness) including increased suicidality [1, 3–6]. These findings appear to be consistent in studies with civilian population and war veterans although the prevalence reported by different studies varies considerably [1, 4, 7]. Prognosis of PTSD also varies with some patients achieving a full recovery and others having a more chronic course [4].

It has been argued that PTSD does not fully capture some of the enduring problems experienced by adults following the exposure to major trauma [8, 9] including high levels of emotional distress and interpersonal dysfunction. Researchers and clinicians working with victims of interpersonal violence and sexual abuse proposed the introduction of two new diagnostic concepts named ‘Disorder of Extreme Stress not otherwise specified’ (DESNOS) and 'complex PTSD' [8, 9]. Although both proposed diagnostic entities were considered for inclusion in DSM-IV [10], this proposal was rejected. Numerous studies with war veterans and victims of torture have reported higher levels of personality pathology in people with PTSD whatever the cause [11–15]. Whilst some people who had been exposed to war trauma had underlying pre-trauma personality related problems, findings from a recent systematic review of extant literature suggested that a proportion of adults with no pre-trauma personality pathology who are exposed to severe trauma appear to go on to develop significant personality problems [16]. Higher levels of exposure to traumatic events have been consistently associated with the increased risk of having a diagnosis of PTSD [17, 18].

In ‘World report on violence and health’ WHO recognises that the violence and cruelty through conflicts are associated with a range of psychological and behavioural problems, including depression, anxiety, suicidal behaviour and PTSD [19]. The WHO experts in the area argue that as long as nations continue to rely on violence to resolve conflicts, this will remain a public health problem and call for more research in this area to gain a better understanding of the effects of interpersonal violence through conflict/war on public mental health [19].

While some previous studies have examined presence of personality pathology in people exposed to war trauma, we found no studies that investigated the severity of traumatic exposure in people who met criteria for personality pathology using a validated personality measure. We therefore set out to investigate whether people who have personality related problems according to the International Personality Disorder Examination (IPDE) screening questionnaire (cases) were more likely to have been exposed to severe war-related trauma (primary exposure) than those who do not have significant personality difficulties (controls). The study was designed to recruit people with personality problems who had lived in a war affected country where many people were directly exposed to war-related activities. We also hypothesized that IPDE positive people would have poorer mental health, a higher use of alcohol and drugs and have higher levels of social dysfunction than controls from the same war-affected region.

## Methods

We conducted a case-control study in which cases met the threshold for having significant personality pathology using the International Personality Disorder Examination (IPDE) screening questionnaire and controls did not. The study was conducted in south Croatia because it is an area that has been affected by repeated military conflicts, most recently during the 1991–1995 war. Followed by the collapse of the former Yugoslavia and subsequent economic crisis, the war could be described as the bloodiest armed conflict in Europe for the last 50 years. It affected several republics of the former Yugoslavia and resulted in several hundred thousand people being killed, over 3 millions being uprooted from their homes with considerable amount of infrastructure being destroyed [20].

### Participants

Both cases and controls were recruited from inpatient and outpatient mental health and general hospital services in Split (southern Croatia). This included inpatient and outpatient mental health services, medical outpatient clinics and several wards at the department of internal medicine. In mental health services, we asked clinicians to refer people who were primarily being treated for personality-related problems, in general hospitals we simply asked to be referred patients who were currently well enough to complete the study interview. The reason for recruiting controls from medical settings was to provide an estimate of the levels of exposure in the same population we recruited cases from: people in contact with healthcare services. To take part in the study potential participants had to have lived in Croatia during the 1991–1995 war and only those who provided written informed consent were recruited. We excluded people suffering from an acute psychotic episode, chronic psychotic illness or from personality change due to organic brain damage, disease and dysfunction. All assessments took place between November 2010 and October 2011. Ethical approval for the study was obtained from the University Hospital Split Ethics Committee and the School of Medicine Ethics Committee, University of Split prior to the start of data collection.

### Measures

The primary outcome measure was the presence of personality disorder (PD) assessed with the 77-item International Personality Disorder Examination (IPDE) screening questionnaire. The IPDE was derived from the original version of Personality Disorder Examination (PDE) and found to have good inter-rater reliability (0.71–0.91) and intertemporal reliability (0.55–0.84) [21, 22]. The IPDE-77 Screening Questionnaire used in this study is a self-report screen containing 77 items written at a 9 years of age reading level that measure personality pathology according to the DSM-IV. The screen has 10 PD subcategories, each containing 7–8 items, except for borderline and narcissistic subgroups which have 9 items. The IPDE requires dichotomous ‘true/false’ responses and the questions are interspersed between different PD subcategories with some items reversed. In this way the likelihood of participants guessing and choosing desirable answers is reduced. We used a more conservative approach for scoring participants answers, so a score of three and below meant ‘negative’ for a PD category and a score of four and above meant ‘positive’ for that personality subgroup. The IPDE screen has been shown to be reliable in clinical and non-clinical populations [23–25].

Traumatic war-related experience and symptoms of post-traumatic stress were assessed by using the Harvard Trauma Questionnaire (HTQ) [26, 27], a self-report measure that has been widely translated and used in traumatised refugees and civilian population, war veterans and victims of torture throughout the world including the communities of the former Yugoslavia [13, 28–30]. The HTQ items related to symptoms of post-traumatic stress are consistent with DSM IV PTSD criteria based on three sub-domains: re-experiencing traumatic events, avoidance and numbing, and increased arousal. A cut off score of ≥2.5, which was initially derived from Indochinese population, was generally considered to be “checklist positive” for PTSD. Although a study conducted in the former Yugoslavia [31] indicated that the cut-off point of ≥2.5 was too high and recommended a cut-off score of ≥2.0 for PTSD ‘positive’ cases, we used the former and a more conservative cut off point of ≥2.5 to reduce the likelihood of making a false positive PTSD diagnosis and to make our findings comparable to wider international communities.

As we were interested in studying the exposure to severe trauma, and in the absence of an established definition of this concept, we have decided to define severe (catastrophic) trauma based on the ICD-10 description of catastrophic stress [32] and the findings from a survey of trauma experts [33]. Our decision to use the ICD-10 classification in this instance (rather than DSM IV) was based on this concept being described in more detail in the ICD 10 which also recognised that personality change may occur following the experience of catastrophic stress [32]. Based on these it was assumed that severe (catastrophic) trauma would involve prolonged exposure to life-threatening circumstances with imminent possibility of being killed (for example exposure to war trauma, concentration camp experience, being tortured, hostage situations and sexual assault). Two authors (JM and MC) independently assessed 47-items of HTQ trauma events (Part I) and selected those items in the HTQ that they thought would meet the criteria for severe trauma. Any disagreements were resolved by further discussions. Out of the 47 war-related traumatic events listed in the Harvard Trauma Questionnaire, 17 items (36%) were considered to be of the severity that could be described as ‘severe’ war-related trauma (according to the above description of catastrophic trauma) and are presented in Table 2.

Symptoms of depression and anxiety were assessed using the Hopkins Symptom Checklist-25 (HSCL-25) [27]. The HSCL-25 is a widely used self-report inventory which has been translated and culturally adapted to different populations across the world including communities of the former Yugoslavia [29]. The HSCL −25 consists of 25 self-report items which are divided into a 10-point anxiety scale and 15-item scale of depressive symptoms that have been experienced in the week prior to the assessment. The ten anxiety symptoms included in the HSCL-25 are consistent with the DSM diagnosis of generalized anxiety disorder, whilst the 15 depression items are applicable to the DSM diagnosis of major depression. We used the recommended cut-off point of ≥1.75 for the HSCL-25 diagnosis of depression and anxiety [27]. The HSCL-25 has been extensively validated in numerous studies on refugees, has high test-retest reliability and good validity in predicting depression and anxiety [29, 30, 34].

The Social Functioning Questionnaire (SFQ) was used to assess participants’ levels of social dysfunction. This is an eight-item self-report measure that was developed from the Social Functioning Schedule [35]. It was found to have good and robust psychometric properties and has been used in a variety of studies and was found to have good test-retest and inter-rater reliability as well as construct validity [36, 37]. The SFQ score of 10 or more indicates poor social functioning and has been found to be positively associated with a diagnosis of personality disorder [36].

Current alcohol and drug misuse were screened by two questions asking participants whether they were using alcohol and drugs (‘yes ‘or ‘no’ answers) at the time of data collection. If the answer was positive to either question, participants were also asked about the type of alcohol/drugs used and weekly amount they consumed.

### Data analysis

Characteristics of the study sample were examined using univariate descriptive statistics. The relationship between categorical explanatory and outcome variables was examined using contingency tables. Differences in proportions were calculated with 95% confidence intervals (CI 95%). The statistical significance of differences was calculated using Chi square (X^2^) tests. Fisher exact test was used if any one cell had an expected frequency of <5.

Standard binary logistic regression was used to examine the relationship between exposure to severe war trauma among cases and controls, controlled for potential confounding effects of other variables (demographic factors). Odds ratios with accompanying 95% confidence intervals were calculated. Caseness (IPDE positive vs IPDE negative) was used as the dependent variable and predictor variables (after being checked for multicollinearity) were entered into a logistic regression model using standard (enter) method.

## Results

In total, 311 patients in mental health and general medical settings were approached for participation in the study of whom 43 (13.8%) individuals refused to take part. 268 participants were included in the study of whom 182 were IPDE positive (cases) and 86 IPDE negative (controls).

Demographic characteristics of cases and controls are presented in the Table 1. There was no significant difference between cases and controls in terms of their age, education, ethnicity or marital status. However, the groups differed in terms of gender as more male than female participants were recruited from the mental health setting (M - 68%; F- 32%) when compared to the general medical setting were similar proportions of male and female participants were recruited (M - 52%; F- 48%).

**Table 1:** Demographic characteristics of participants who met and did not meet IPDE threshold for personality disorder diagnosis

Variable	Cases (IPDE positive) *N* = 182	Controls (IPDE negative) *N* = 86	Mean or proportion difference (95% CI of the difference)
Age - Mean (SD)	45.14 (9.65)	46.64 (12.15)	-1.49 (−4.45 to 1.45)
Gender *N* (%)			
Male	123 (67.6)	45 (52.2)	0.15 (0.03 to 0.27)
Female	59 (32.4)	41 (47.7)	
Ethnicity *N* (%)			
Croatian	176 (96.7)	84 (97.7)	0.01 (−0.05 to 0.05)
Bosnian	3 (1.6)	0 (0.0)	0.02 (−0.03 to 0.05)
Serbian	1 (0.5)	1 (1.2)	0.01 (−0.02 to 0.06)
Montenegrin	0 (0.0)	1 (1.2)	0.01 (−0.01 to 0.06)
Macedonian	1 (0.5)	0 (0.0)	0.01 (−0.04 to 0.03)
Other	1 (0.4)	0 (0.0)	0.01 (−0.04 to 0.03)
Education (%)			
No qualifications	16 (8.8)	14 (16.3)	0.07 (−0.005 to 0.17)
A levels/vocational	132 (72.5)	52 (60.5)	0.12 (0.002 to 0.24)
University	34 (18.7)	20 (23.3)	0.05 (−0.05 to 0.16)
Marital status *N* (%)			
Married	112 (61.5)	61 (70.9)	0.09 (−0.03 to 0.21)
Divorced	9 (4.9)	1 (1.2)	0.04 (−0.02 to 0.08)
Separated	10 (5.5)	3 (3.5)	0.02 (−0.05 to 0.07)
Single	39 (21.4)	16 (18.6)	0.03 (−0.08 to 0.12)
Widowed	3 (1.6)	2 (2.3)	0.01 (−0.03 to 0.06)
Living with partner	9 (4.9)	3 (3.5)	0.01 (−0.05 to 0.06)
Recruitment area *N* (%)			
Outpatient	175 (96)	77 (90)	0.07 (0.01 to 0.15)
Inpatients	7 (4)	9 (10)	

### Trauma exposure

Among the 182 cases, 169 (92.9%) reported being exposed to one or more war related events as did 38 (44.2.%) of controls (X^2^ = 78.69, *p* < 0.001) indicating that cases were twice as likely to report an event on the HTQ than controls. Regarding exposure to severe war-related trauma, 72.5% of cases and 24.4% of controls experienced one or more severe war-related events (OR = 8.17, 95% CI 4.53 to 14.74). The association between severe war-related events and IPDE caseness increased further after adjusting for gender and marital status and then for all demographics, with the odds of being 10 times higher for those undergoing a severe war-related event (OR = 10.1, 95% CI 5.0 to 20.4).

Comparisons of different types of severe war-related traumatic events in cases and controls based on 17 selected items from the Harvard Trauma Questionnaire are presented in Table 2. There was a statistically significant difference between the two groups on 10 out of 17 traumatic events indicating that a higher proportion of cases were exposed to severe war-related traumatic events. When we looked into the type of severe traumatic events among those 10 HTQ items, results indicated that 50% of events were related to participants own life being in danger and 50% of them involved participants witnessing extreme violence inflicted on others or the result of violence towards others (such as murder, torture, seeing burned or disfigured bodies). There was no significant difference between the two groups on levels of interpersonal violence affecting close family members (spouse or children).

**Table 2:** Comparisons of catastrophic trauma events (based on HTQ) between cases and controls

Type of severe traumatic event (based on Harvard Trauma Questionnaire)	Cases (IPDE positive) *N* = 182	Controls (IPDE negative) *N* = 86	Proportion difference (95% CI of the difference)
Being under sniper fire *N* (%)	100 (55.2)	11 (12.8)	0.42 (0.31 to 0.51)**
Witnessed burned or disfigured bodies *N* (%)	85 (47.0)	7 (2.6)	0.39 (0.28 to 0.47)**
Murder or death due to violence of other family members (not spouse or a child) or friends *N* (%)	58 (32.2)	8 (9.3)	0.23 (0.12 to 0.31)**
Ran into an ambush *N* (%)	62 (34.1)	3 (3.5)	0.31(0.22 to 0.38) **
Witnessed torture *N* (%)	33 (18.1)	1 (1.2)	0.17 (0.10 to 0.23) **
Witness killing or murder *N* (%)	33 (18.3)	0 (0)	0.18 (0.12 to 0.24) **
Serious physical injury from combat or landmine *N* (%)	24 (13.3)	1 (1.2)	0.12 (0.05 to 0.18) **
Other types of sexual abuse or sexual humiliation (excluding rape) *N* (%)	11 (6.1)	0 (0.0)	0.06 (0.01 to 0.11)*
Torture *N* (%)	11 (6.1)	0 (0.0)	0.06 (0.01 to 0.11)*
Forced to find and burry bodies of the dead *N* (%)	10 (5.5)	0 (0.0)	0.06 (0.01 to 0.09)*
Solitary confinement *N* (%)	9 (4.9)	0 (0.0)	0.05 (0.001 to 0.09)
Forced to harm others *N* (%)	9 (4.9)	0 (0.0)	0.05 (0.001 to 0.09)
Murder or death due to violence of son or daughter N (%)	3 (1.6)	2 (2.3)	0.01 (−0.03 to 0.06)
Rape *N* (%)	2 (1.1)	0 (0.0)	0.01 (−0.03 to 0.04)
Kidnapped *N* (%)	1 (0.5)	1 (1.2)	0.01 (−0.03 to 0.04)
Murder or death due to violence of spouse *N* (%)	2 (1.1)	0 (0.0)	0.01 (−0.02 to 0.06)
Witnessed rape or sexual abuse *N* (%)	2 (1.1)	0 (0.0)	0.01 (−0.03 to 0.04)

* *p* < 0.05 X^2^ test for difference in proportions with 1 degree of freedom

** *p* < 0.001 X^2^ test for difference in proportions with 1 degree of freedom

### Comparisons of health and social functioning between groups

Univariate analysis showed that IPDE positive participants reported significantly more depression, anxiety and PTSD symptoms. They also had more interpersonal dysfunction with mean score of 12.58 (sd = 3.90), compared to a mean score of 4.62 (sd = 2.83) among IPDE negative participants, t(254) = 16.45, *p* < 0.001, mean difference 7.96 (95% CI = 7.11–8.81).

Variables related to mental health and social functioning were reclassified as dichotomous variables (i.e. met criteria for depression vs did not meet criteria for depression) and used in subsequent multivariate analysis. Depression and anxiety were highly correlated (>0.8), therefore only depression was included in further analysis to minimise risk of multicollinearity. Table 3 displays the crude and adjusted odds ratios with 95% confidence intervals. A greater proportion of people in the IPDE screen positive group met criteria for depression and PTSD caseness and reported higher levels of social dysfunction after controlling for gender and educational attainment.

**Table 3:** Comparisons of mental health and social function between cases and controls

Variables	Cases	Controls	OR (95% CI)	Adjusted OR (95% CI) (gender and educational level)
Depression *N* (%)	165 (90.7)	17 (19.8)	4.37 (1.64 to 11.66) *	4.70 (1.67 to 13.24) *
PTSD *N* (%)	102 (62.2)	2 (2.5)	7.52 (1.42 to 39.85)*	7.80 (1.38 to 43.93)*
Social dysfunction, SFQ >10 *N* (%)	136 (77.7)	6 (7.4)	10.39 (3.51 to 30.75)**	11.75 (3.77 to 36.54)**

*p < 0.05

**p < 0.001

Similar proportions of cases and controls reported current use of alcohol (39.8% vs. 37.2%). However, the alcohol consumption in cases was significantly higher with a mean of 14.24 units per week (sd = 11.03) when compared to controls whose mean number of alcohol units was 9.24 (sd = 7.25), t (73) = 2.16, *p* < 0.05, mean difference 4.99 (95% CI = 0.39 to 9.60). When the groups were compared on weekly alcohol consumption, a significantly higher number of cases were drinking above 20 units of alcohol per week (X^2^ (1, *n* = 268) = 6.46, *p* = 0.04), although the proportion of them was relatively low (7.1%). Around 13% of cases failed to disclose the amount of weekly alcohol consumption. Similarly, a significantly higher number of cases reported current substance misuse (8.2% vs. 0.0%) X^2^ (1, *n* = 268) = 7.51, *p* < 0.05).

## Discussion

When we compared study participants who had significant personality pathology according to the IPDE screen (cases) to those who did not have personality-related problems (controls), the results indicated that cases were almost twice as likely as controls to report at least one war-related event on the HTQ trauma event scale. However, when the war-related events on the HTQ scale were reclassified according to the severity to those considered to be of a severe (catastrophic) nature and those that were not, our findings indicated that IPDE positive people were eight times more likely to have been exposed to severe war-related trauma than IPDE negative participants. This association increased further after adjustment for demographic factors. These results are consistent with our primary hypothesis that people who have clinically significant personality pathology were more likely to have been exposed to war-related trauma (primary exposure) than those who did not have personality-related problems.

When considering different types of severe trauma according to the war-related event scale on the HTQ two distinctive types of traumatic events dominated. One group of events involved participants’ own life being in direct danger (for example being under sniper fire) and the second group of events involved participants witnessing extreme violence inflicted on others or the result of violence towards others. Although both cases and controls reported the aforementioned traumatic events, a significantly higher proportion of cases were exposed to severe traumatic events.

We found a strong association between exposure to severe war-related trauma and personality pathology. These findings could be explained – in part or in whole by ‘reverse causation’ i.e. that people with personality disorder due to their vulnerability were more likely to be exposed to severe war trauma. However, there is a possibility that a proportion of participants in this study developed interpersonal dysfunction after exposure to severe war trauma i.e. in adulthood. In other words, it is possible that IPDE caseness observed in some participants in the study has emerged secondary to severe trauma. This would be consistent with the findings from a recent systematic review which suggested that a proportion of adults with no pre-trauma personality pathology appear to go on to develop significant personality problems following the exposure to severe trauma [16]. However, one would need to demonstrate that severe war trauma antedated the outcome (personality pathology) before the observed association could be viewed as evidence for causation which emphasizes the importance of Hill’s temporality criterion [38]. The findings also suggest that participants had personality related problems and interpersonal dysfunction 15 years after the war. In view that the population examined in this study comes from the people in contact with healthcare services, it is possible that these results may not apply to the general population, thus affecting the generalizability of the findings.

### Comparison of mental health and social functioning between cases and controls

As expected, people who were IPDE positive had poorer mental health and social functioning than controls indicating more global impairment. A significantly higher proportion of them met criteria for depression caseness and had higher levels of social dysfunction. These findings are consistent with prior research which reported considerable comorbidity and increased levels of social dysfunction in patients with personality disorders [39–41].

People who were IPDE positive were also more likely to report having PTSD symptoms compared to controls. This is likely to be explained by higher levels of exposure to war-related adverse events in cases than controls and is consistent with previous research from war-affected areas of the Balkan states [2, 42].

Alcohol and drug use were higher in cases than controls. These findings are consistent with evidence from prior research which suggests higher levels of alcohol and illicit substance abuse in PTSD and PD patients [4, 43, 44]. Although both alcohol and drug misuse were relatively low in this study, these findings are consistent with low levels of substance misuse also found in a cross-sectional study examining mental disorders in a post-war adult population sample in the Balkan countries [2].

### Strengths and limitations

There are several strengths to this study. It is the only study to our knowledge, that is addressing Axis II disorders and the severity of trauma in individuals exposed to war-related trauma. Trauma exposure was assessed consistently using a self-report measure that has been translated, culturally adapted and extensively used, tested and validated in the communities of former Yugoslavia. An additional strength to this study is that it was conducted in a war-affected area which allowed recruitment of a large number of participants with exposure to war trauma (the primary exposure of interest). Multivariate analysis was used to avoid undesirable effects of potential confounders, a justified approach when controlling of confounders is not fully feasible in the study design itself [38].

However, there are also a number of limitations. The most important one is recall bias, which could have influenced the participants’ accounts of traumatic war experience. Recall bias can be minimised if exposure status between cases and controls is assessed in a sufficiently similar way which in this study was done by using a standardised self-report measure to assess trauma exposure (HTQ) in cases and controls ensuring that the exposure of interest (war trauma) was equally assessed in both groups. Another limitation of the study is related to the use of self-report measures to assess personality pathology. Diagnostic value of personality screening questionnaires has been questioned when compared to semi-structured interviews [45, 46]. However, the IPDE screen has been used to assess personality pathology in the WHO survey including 13 widely distributed countries and some other studies and reportedly provided consistent and reliable results [47, 48]. Additionally, in our study most of the cases recruited already had a clinical diagnosis of personality disorder. A further limitation of the study is the lack of collateral history to validate participants’ accounts of interpersonal dysfunction. Finally, a caution is needed when interpreting the generalizability of the findings in view that the population examined comes from the people in contact with healthcare services, although one could argue that it is likely that what holds true for people who have contact with healthcare services holds true for those that do not.

## Conclusion

Findings from this study suggest that in addition to short and medium-term impact of war, there are long-term consequences on mental health, interpersonal and social functioning which have implications for assessing and meeting the long-term mental health needs of people in war-affected regions. Future research is needed to examine what proportion of post-traumatic personality pathology is truly a result of severe traumatic war exposure rather than pre-existing PD.

## Abbreviations

HTQ: Harvard trauma questionnaire
HSCL-25: Hopkins Symptom Checklist-25
IPDE: International Personality Disorder Examination
PTSD: Post-traumatic stress disorder
SFQ: Social Functioning Questionnaire
